# Intelligent Detection of Tomato Ripening in Natural Environments Using YOLO-DGS

**DOI:** 10.3390/s25092664

**Published:** 2025-04-23

**Authors:** Mengyuan Zhao, Beibei Cui, Yuehao Yu, Xiaoyi Zhang, Jiaxin Xu, Fengzheng Shi, Liang Zhao

**Affiliations:** 1 School of Artificial Intelligence and Big Data, Henan University of Technology, Zhengzhou 450001, China; zhaomy@stu.haut.edu.cn; 2 College of Electrical Engineering, Henan University of Technology, Zhengzhou 450001, Chinazhaoliang_270@163.com (L.Z.)

**Keywords:** tomato, ripeness detection, YOLO-DGS, C2f-GB, BiFPN, channel attention

## Abstract

To achieve accurate detection of tomato fruit maturity and enable automated harvesting in natural environments, this paper presents a more lightweight and efficient maturity detection algorithm, YOLO-DGS, addressing the challenges of subtle maturity differences between regular and cherry tomatoes, as well as fruit occlusion. First, to enhance feature extraction at various levels of abstraction in the input data, this paper proposes a novel segment-wise convolution module, C2f-GB. This module performs convolution in stages on the feature map, generating more feature maps with fewer parameters and computational resources, thereby improving the model’s feature extraction capability while reducing parameter count and computational cost. Next, based on the YOLO v10 algorithm, this paper removes redundant detection layers to enhance the model’s ability to capture specific features and further reduce the number of parameters. This paper then integrates a bidirectional feature pyramid network (BiFPN) into the neck network to improve feature capture across different scales, enhancing the model’s ability to handle objects of varying sizes and complexities. Finally, we introduce a novel channel attention mechanism that allows the network to dynamically adjust its focus on channels, efficiently utilizing available information. Experimental results demonstrate that the improved YOLO-DGS model achieves a 2.6% increase in F1 score, 2.1% in recall, 2% in mAP50, and 1% in mAP50-95. Additionally, inference speed is improved by 12.5%, and the number of parameters is reduced by 26.3%. Compared to current mainstream lightweight object detection models, YOLO-DGS outperforms them, offering an efficient solution for the tomato harvesting robot vision system in natural environments.

## 1. Introduction

Tomatoes are a significant crop, with global production exceeding 180 million tons [1]. However, due to their susceptibility to skin damage, wilting, and storage challenges [2], they are primarily supplied to local and nearby markets. To improve the global coverage of the tomato supply chain and ensure efficient and safe transportation to domestic and international markets while maintaining quality, it is essential to select the appropriate ripeness stage based on transportation distance and storage duration at harvest [3]. Although tomato harvesting robot systems have significantly improved the success rate and efficiency of continuous harvesting [4], there are still limitations in the accuracy of ripeness detection. Developing algorithms for automated ripeness detection with high recognition rates is crucial for optimizing the distribution of tomatoes at different ripeness levels and enhancing harvesting automation efficiency [5].

**Figure 1:**
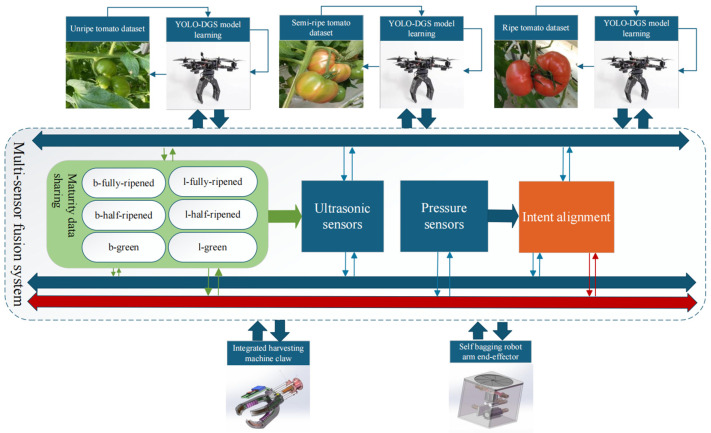
Architecture of the multi-sensor fusion system.

Significant advancements have been made in the application of drones within the fields of artificial intelligence and machine learning, particularly in smart agriculture. In the field of computer vision, drones can capture high-resolution images and videos, facilitating advanced functions such as image recognition, object tracking, and even 3D scanning [6,7]. As illustrated in Figure 1, an intelligent tomato harvesting system designed for smart agriculture demonstrates the power of multi-sensor fusion technology [8,9,10] and deep learning algorithms to enable fully automated operations [11]. At the core of this system lies the YOLO-DGS object detection model, which was trained on a comprehensive tomato dataset encompassing various maturity levels, including semi-ripe and fully ripe stages. This model is capable of accurately identifying six distinct maturity stages, such as b-fully-ripened and l-half-ripened fruits. In terms of fruit harvesting, existing methods primarily combine manual bagging and manual picking, which rely on human labor and are limited by high labor costs and low harvesting efficiency [12,13,14]. In the perception layer, the system integrates ultrasonic sensors for real-time distance measurement and obstacle avoidance, alongside pressure sensors to monitor harvesting force. These sensors, combined with visual recognition results, form a multi-dimensional perception network. The actuators feature an adaptive robotic end-effector [15] that intelligently adjusts grasping force and angle based on fruit maturity and real-time sensor feedback, ensuring precise and damage-free harvesting. This system establishes a complete closed-loop workflow, seamlessly integrating environmental perception, fruit recognition, maturity assessment, and automated harvesting. It exemplifies the synergistic application of machine vision, multi-sensor fusion [16], and intelligent control technologies in modern smart agriculture, offering an efficient and reliable solution for automated agricultural produce harvesting [17]. The promotion of smart agriculture has also reduced the physical labor required of farmers and lowered the overall costs, bringing favorable opportunities for the development of agriculture [18].

In recent years, scholars have conducted extensive research to address the challenges in fruit maturity detection. Firstly, for the image detection challenges in fruit maturity assessment, from 2019 to 2021, early approaches focused on basic detection frameworks. Liu et al. (2020) [19] pioneered multi-level deep residual networks for tomato maturity recognition, achieving high precision but requiring intensive computational resources. Hsieh et al. (2021) [20] integrated R-CNN with binocular imaging, demonstrating accurate localization in controlled greenhouse conditions but with accuracy decreasing under different light conditions. From 2021 to 2023, advancements addressed environmental robustness; Zu et al. (2021) [21] implemented Mask R-CNN for all-weather green tomato detection, yet performance decreased with overlapping fruits. Li et al. (2023) [22] developed MHSA-YOLOv8 for tomato counting, but in occluded scenes the F1 score was reduced. From 2024 to 2025, current solutions emphasize complex scenarios; Ji et al. (2024) [23] introduced apple picking sequence planning, but dense foliage caused part of the path planning to fail. Dong et al. (2025) [24] enhanced GPC-YOLO for unstructured environments, maintaining higher accuracy under occlusion but requiring higher power consumption. These studies have several core limitations, including a reduction in accuracy across methods due to severe occlusion, performance differences under light variations, and high computational costs that limit real-time applications. Secondly, for the algorithm deployment challenges, from 2022 to 2023, Zhou et al. [25] combined YOLOv7 with classical image processing, improving positioning accuracy but requiring higher RAM for deployment. Zeng et al. [26] created a mobile-optimized YOLO variant, yet model pruning caused mAP drop. From 2024 to 2025, Li et al. (2024) [27] developed lightweight YOLOv5s for pitaya detection, but nighttime operations needed supplemental lighting. Wang et al. [28] improved RT-DETR for tomato ripeness, though the ARM processor had high latency. Ji et al. (2025) [29] incorporated Transformer modules, increasing model size and presenting challenges for edge devices. Thirdly, for the multi-sensor fusion approaches, Mu et al. [30] demonstrated occluded tomato detection using RGB-D fusion, but it required more power consumption versus monocular systems. Li et al. [31] advanced with dual-frequency LiDAR, but processing delays were greater. Wang et al. (2024) [32] combined R-LBP with YOLO-CIT, improving citrus detection but requiring calibrated lighting.

In summary, it can be concluded that single-stage object detection algorithms based on neural networks have become mainstream in the field of fruit maturity detection. Although these efficient and accurate methods often involve high model complexity, resulting in slower computation and detection speeds, lightweight and fast detection methods tend to sacrifice accuracy [33,34]. In resource-constrained environments, achieving an ideal balance between speed, accuracy, and computational resource consumption to meet real-time detection requirements for industrial applications, while ensuring high-precision results and maintaining model efficiency, remains a significant challenge. Particularly in tomato maturity detection, the inconsistency in maturity within the same inflorescence and the severe occlusion scenarios prevalent in greenhouse environments can interfere with the recognition process. To address these issues, this paper proposes a lightweight model, YOLO-DGS (D-2Detect, G-C2f-GB, S-Squeeze-and-Excitation), based on an improved YOLO v10 algorithm. This method (1) introduces a novel segmented convolution calculation module, C2f-GB, which merges the Channel to Feature (C2f) module with GhostBottleneck. This module performs convolutional calculations on feature maps in stages, using fewer parameters and computations to generate more feature maps, thereby enhancing feature extraction while reducing parameter volume and computational complexity. (2) Reduces redundant detection layers in YOLO v10, improving the model’s ability to capture specific features and lowering parameter volume. (3) Integrates a bidirectional feature pyramid network (BiFPN) in the neck network, which improves traditional FPN’s information fusion by adding contextual information and assigning corresponding weights, thereby enhancing the expressiveness of the feature pyramid. (4) Incorporates a channel attention mechanism (Squeeze-and-Excitation) in the neck network to effectively detect different tomato maturities.

This paper is organized into five sections. To highlight the innovations of this study, Section 1 summarizes previous research in the field. Section 2 describes the enhanced network architecture designed for more lightweight and precise fruit maturity detection. Section 3 presents an analysis of the results to evaluate the detection network’s performance. Finally, Section 4 provides the conclusion.

## 2. Materials and Methods

### 2.1. Network Framework

**Figure 2:**
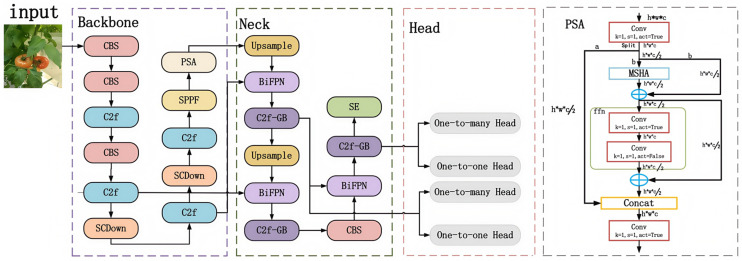
Structure of the YOLO-DGS model. * represents multiplication.

The accuracy, speed, and lightweight nature of the tomato detection model are crucial for the efficient operation of harvesting robots [35]. Addressing the multi-scale target detection characteristics in complex backgrounds, this paper proposes a lightweight target detection algorithm, YOLO-DGS, whose structure is shown in Figure 2. The model is an improvement upon YOLO v10, with a primary focus on lightweight design in the neck network. First, this paper removes the maximum target detection layer in the YOLO v10 detection head to reduce the model’s width, depth, and parameter count, thereby enhancing its target recognition capability. Second, we introduce the C2f-GB, a segmented convolution module that performs staged convolution on feature maps, significantly reducing both parameter and computational load while maintaining accuracy. Additionally, the BiFPN (bidirectional feature pyramid network) is adopted to more effectively transmit and integrate high- and low-level features. Finally, this paper incorporates the SE attention mechanism (Squeeze-and-Excitation Networks) to explicitly model the interdependencies between convolutional feature channels, thus enhancing the network’s representational power.

### 2.2. C2f-GB Module

**Figure 3:**
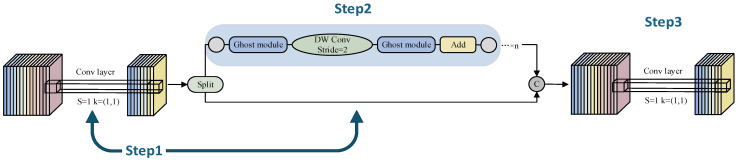
Structure of the C2f-GB model.

The original C2f module employs a stacked convolutional bottleneck structure, which has high computational complexity. Due to consecutive convolutional operations, the deep-level processing path may lose shallow-layer details. This increases the model’s parameters and computational cost, making it unsuitable for lightweight applications. To address these issues, we designed a novel segmented convolutional network, C2f-GB. In the first step, a regular convolution is performed, followed by data splitting. In the second step, a “phantom feature” [36] generation mechanism is adopted, where redundant convolutions are replaced with low-cost linear operations to reduce computational and parameter burdens. In the third step, the convolution results from the first and second steps are combined by adding their channels, and then another regular convolution is carried out. This approach not only alleviates the computational pressure but also enhances feature retention, explicitly fusing multi-level information and preventing the loss of shallow-layer details. As a result, even after lightweight optimization, the model can maintain high representational ability, making it suitable for real-time tasks in resource-constrained environments. Figure 3 illustrates the workflow of C2f-GB. The segmented convolution is mainly reflected in the first two steps. In the first step, an initial convolution (cv1) extracts features and adjusts the number of channels to generate intermediate feature maps. These maps are then split into two parts along the channel dimension. One part directly proceeds to the subsequent concatenation step, preserving the original features. The other part enters a processing chain composed of multiple GhostBottleneck modules in the second step. Each module consists of the following: The first Ghost module generates a part of the feature map through a small standard convolution and then creates “phantom” feature maps using low-cost operations. These two types of feature maps are concatenated to expand the number of channels. The second Ghost module compresses the number of channels to match the input, using depth-wise separable convolutions to reduce computational cost. A residual connection ensures effective gradient flow and mitigates network degradation. After processing, part B and part A are concatenated along the channel dimension, merging detailed and deep-level features. Finally, a convolution (cv2) adjusts the number of channels and integrates the information to produce the final output.

**Figure 4:**
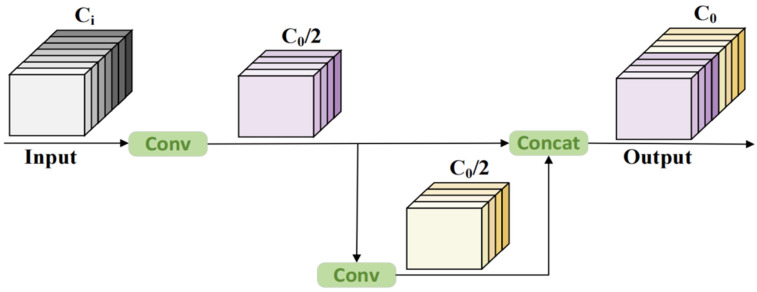
Architecture of the Ghost module.

The GhostBottleneck module plays a critical role in C2f-GB by generating redundant features through inexpensive operations, significantly reducing parameters and computational complexity compared to traditional convolutions. This makes it more suitable for mobile and edge devices. Similar to the basic residual block in ResNet, the GhostBottleneck module combines multiple convolutional layers with shortcuts. The C2f-GB network consists of two stacked Ghost modules: the first serves as an expansion layer to increase the channel count, with an expansion ratio that reflects the output-to-input channel ratio; the second Ghost module reduces the channel count to match the shortcut path. These modules are connected via a downsampling layer and depthwise convolution with a stride of 2. Inspired by MobileNetV2, the second Ghost module in C2f-GB does not apply ReLU after depthwise convolutions to avoid information loss. Batch normalization (BN) and ReLU activation are applied after each layer. The operation of the stacked Ghost modules is implemented through stepwise convolutions, as shown in Figure 4. The theoretical acceleration ratio of Ghost modules compared to regular convolutions is as follows: (Among them, *c* represents the input channels, *n* represents the output channels, *h*′ and *w*′ are the height and width of the feature map, respectively. The size of the conventional convolutional kernel is *k*, the size of the convolutional kernel for the linear transformation is *d*, and the transformation is carried out *s* times).

(1) $$r_{s}=\frac{n\cdot h^{\prime }\cdot w^{\prime }\cdot c\cdot k\cdot k}{\frac{n}{s}\cdot h^{\prime }\cdot w^{\prime }\cdot c\cdot k\cdot k+(s-1)\cdot \frac{n}{s}\cdot h^{\prime }\cdot w^{\prime }\cdot d\cdot d}=\frac{c\cdot k\cdot k}{\frac{1}{s}\cdot c\cdot k\cdot k+\frac{s-1}{s}\cdot d\cdot d}\approx \frac{s\cdot c}{s+c-1}\approx s$$

### 2.3. Small Object Detection

The original YOLO v10 model includes three detection layers for detecting large, medium, and small targets [37]. However, in this dataset, the primary targets—normal and cherry tomatoes—are medium to small in size, making the maximum target detection layer less effective. To address this, this paper removed the maximum target detection layer from the neck network and detection head, enhancing the model’s ability to capture specific features of normal and cherry tomatoes while reducing the model’s depth, width, and parameters to improve inference speed. To further validate the effectiveness and feasibility of this improvement, this paper conducted a comparative analysis with different numbers of detection heads. The results are shown in Table 1.

Table 1 indicates that, compared to models with one, three, and four detection heads, the improved two-detection-head model (YOLO-DGS) achieved 3.5%, 0.3%, and 3.1% improvements in recall rate, respectively. The detection speed increased by 59.6 fps, 71.9 fps, and 153.5 fps, respectively. Compared to models with three and four detection heads, the parameter count decreased by 26.7% and 32.2%, respectively. Furthermore, the mAP50 increased by 3.1% and 1.4% when compared to models with one and four detection heads. Although the mAP50 for the three-detection-head model is slightly higher, the other performance metrics of the improved model surpass those of the three-detection-head configuration. This clearly demonstrates that removing the maximum target detection layer improves target recognition in this dataset and yields better overall performance.

### 2.4. Bi-Directional Feature Pyramid Network

**Figure 5:**
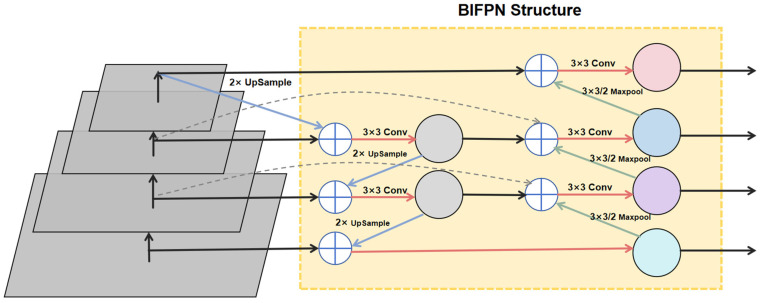
BiFPN architecture diagram.

The Bi-Directional Feature Pyramid Network (BiFPN), based on the FPN network, enhances the feature pyramid’s expressive power by adding contextual information and assigning appropriate weights to fuse feature information. BiFPN introduces bidirectional connections between adjacent levels of the feature pyramid during feature fusion, enabling bidirectional information flow. The enhanced network performs feature integration from high-level to low-level features (top-down) and low-level to high-level features (bottom-up). This bidirectional feature integration helps more effectively capture information across different scales, improving the tomato ripeness detection network’s ability to handle objects of various sizes and complexities. BiFPN also uses a weighted feature fusion mechanism, optimizing multi-scale feature integration through adaptive learning of fusion weights, allowing the network to more effectively leverage features from different layers, as shown in Figure 5.

### 2.5. Optimized Channel Attention Mechanism

**Figure 6:**
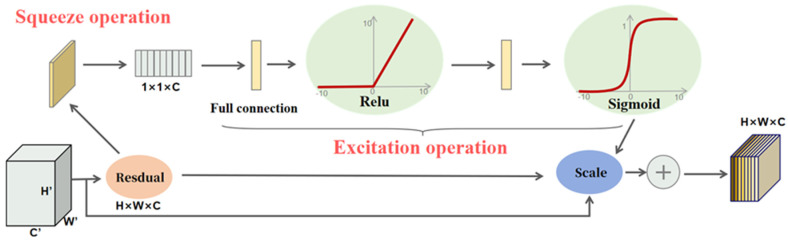
Squeeze-and-Excitation network structural diagram.

The Squeeze-and-Excitation (SE) mechanism introduces a channel attention mechanism, as depicted in Figure 6, enabling the neural network to focus on the most crucial features for the current task. The core operations of the SE module consist of the squeeze operation and the excitation operation. Firstly, given an input feature map *X*∈*R^H^*^×*W*×*C*^, the squeeze operation aggregates the feature map across the spatial dimensions (*H* × *W*) through global average pooling (GAP) to generate a channel descriptor. As shown in Equation (2), where *z_c_* is the compressed scalar value of the *c*-th channel, *H* and *W* are the height and width of the input feature map, respectively, and *x_c_*(*i*,*j*) is the feature value at the spatial position (*i*, *j*) of the *c*-th channel of the input feature map. That is, for an input feature map with dimensions *H*×*W*×*C*, the global average pooling operation calculates the average value of each channel across all spatial positions, compressing each channel into a single scalar. The generated average values form a vector of length *C*, which serves as the channel descriptor of the feature map. Next, the excitation operation applies a non-linear transformation to the channel descriptor *z* to generate channel weights, as shown in Equation (3). Here, s is the final generated vector of channel weights, *W*_1_ is the weight matrix of the first fully connected layer, which is used for dimensionality reduction, *W*_2_ is the weight matrix of the second fully connected layer, which is used to restore the dimension, *δ* represents the ReLU activation function, introducing non-linearity, and *σ* is the Sigmoid activation function, which restricts the weights within the range of [0, 1]. This operation applies a weighting function to the channel descriptor obtained from the squeeze operation to emphasize the important channels. Specifically, the C-dimensional vector obtained from the squeeze operation passes through a fully connected (FC) layer, which introduces a non-linear transformation via an activation function (such as ReLU). The weights learned by the FC layer are restricted to the range between 0 and 1 by the Sigmoid activation function. These learned weights reflect the activation level of each channel. Finally, the original C-dimensional vector is multiplied by these weights to generate a weighted vector, as shown in Equation (4). Here, the result is the weighted output feature map, which dynamically adjusts the attention of each channel, enhancing the network’s ability to efficiently utilize information.

(2) $$z_{c}=\frac{1}{H\times W}\sum_{i=1}^{H}\sum_{j=1}^{W}x_{c}(i,j)$$

(3) $$s=\sigma (W_{2}\cdot \delta (W_{1}z))$$

(4) $$\overset{\wedge }{X}=s\cdot X$$

## 3. Experiment

### 3.1. Dataset

**Figure 7:**
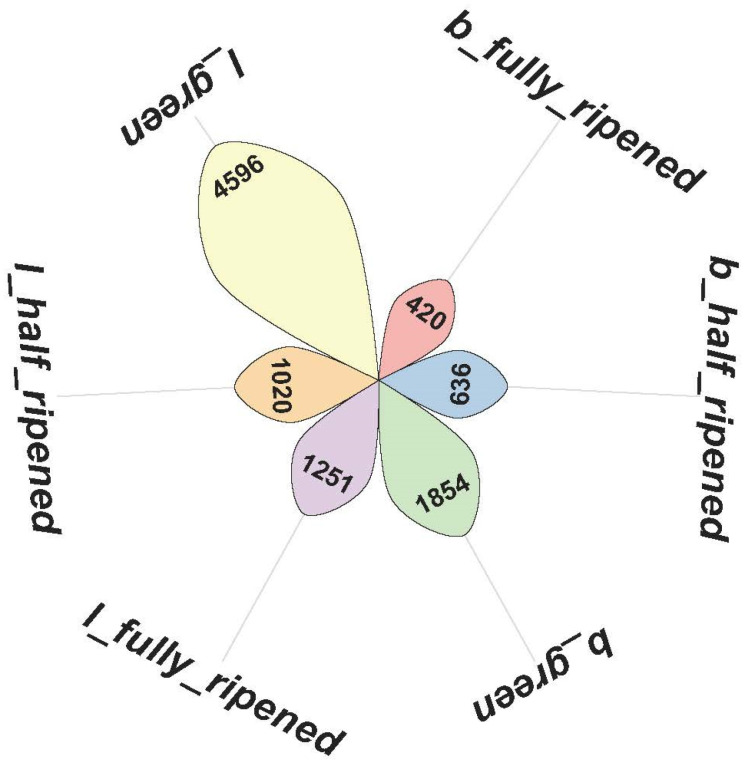
Distribution of target counts.

**Figure 8:**
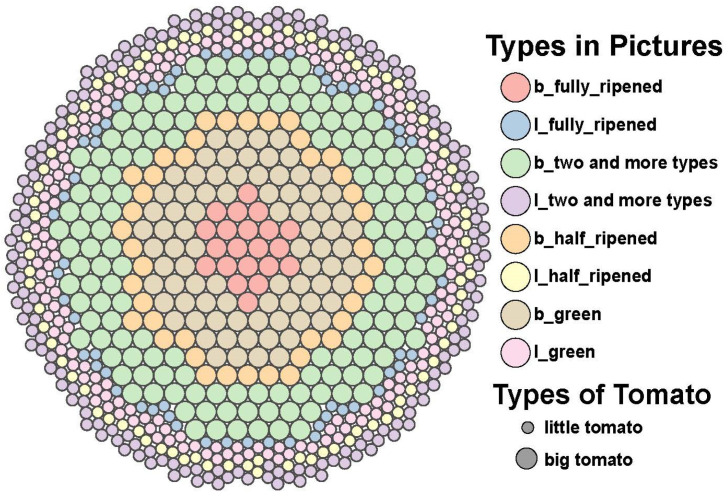
Distribution of target types in the image.

**Figure 9:**
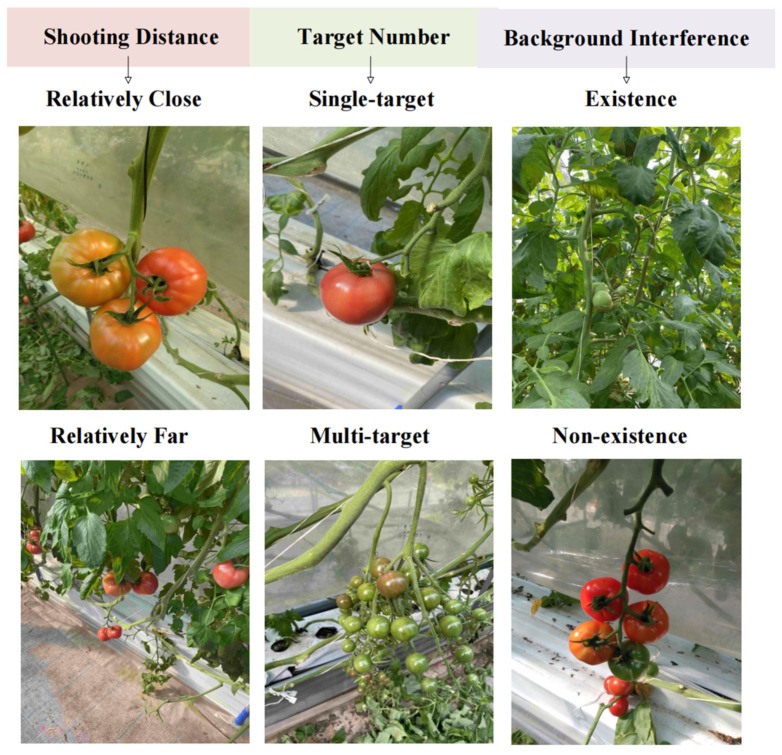
Different cases of datasets.

This experiment focuses on two types of tomatoes: common tomatoes and cherry tomatoes, with each type having three maturity stages: ripe, semi-ripe, and unripe. The dataset used in this study is sourced from a publicly available tomato fruit dataset from local farms in Chennai, Tamil Nadu, India. This dataset comprises 804 images of common tomatoes and cherry tomatoes, with a total of 9777 annotated targets. Figure 7 illustrates the distribution of each target category, while Figure 8 shows the types of targets present in each image. In Figure 8, the larger circles represent common tomatoes, and the smaller circles represent cherry tomatoes. The dataset was captured using an independent camera, and the images have a resolution of 3024 × 4032. In terms of the shooting distance, the dataset includes two types: relatively close and relatively far. Correspondingly, the sizes of the target objects are also classified into larger and smaller according to the distance. Regarding the number of targets, the dataset covers two situations: single-target and multi-target. As for the background interference, the dataset is divided into two cases: with background interference and without background interference, as shown in Figure 9. The dataset was randomly divided into a training set, a validation set, and a test set, with a ratio of 7:2:1. The weight decay was set to 0.0005, and the initial learning rate was set to 0.01.

### 3.2. Experimental Setting

**Figure 10:**
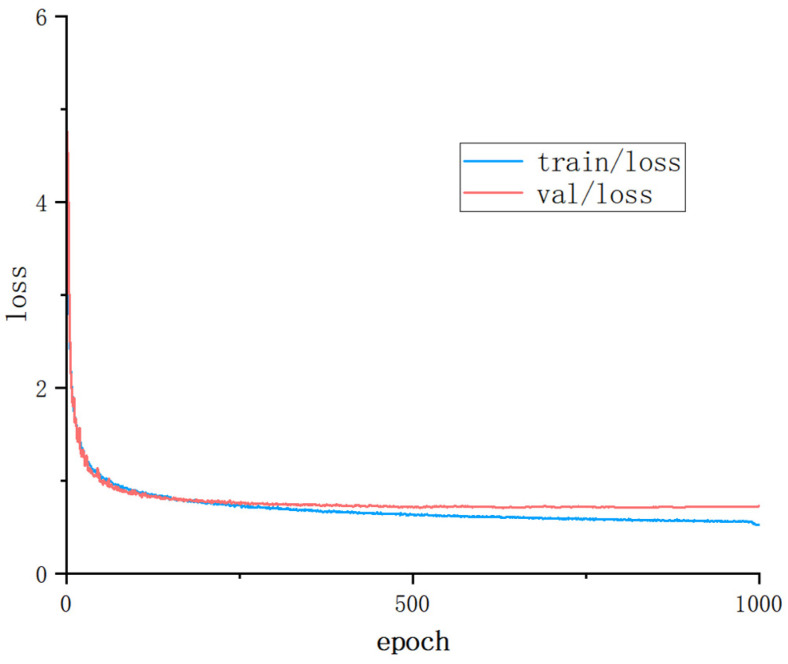
Loss curves for training and validation sets.

To ensure the effectiveness of the experiments, all the experiments presented in this work were conducted in a consistent operating environment. The specific configurations are detailed in Table 2. In order to obtain the best training results, all the models in this experiment were trained for 1000 epochs. The loss curves of the training set and the validation set are shown in Figure 10. As can be seen from the figure, the trends of the train/loss and the val/loss are consistent. Both of them decrease as the number of epochs increases. The loss of the validation set is always slightly higher than that of the training set, but there is no divergence phenomenon. Moreover, the gap between the two is within a reasonable range, which is the normal generalization error of the model on the validation set. Therefore, the model does not exhibit the situation of overfitting.

### 3.3. Evaluation Metrics

The evaluation metrics used in this study include Precision (*P*), Recall (*R*), *F*1 Score, mean Average Precision (m*AP*), the number of parameters (in megabytes), and inference speed in Frames Per Second (FPS). The formulas for these metrics are as follows:

(5) $$P=\frac{TP}{TP+FP}$$

(6) $$R=\frac{TP}{TP+FN}$$

(7) $$F1=\frac{2\times P\times R}{P+R}$$

(8) $$mAP=\frac{1}{n}\sum_{i=1}^{n}AP_{i}$$

where *TP* represents the number of true-positive samples, *FP* represents the number of false-positive samples, *FN* represents the number of false-negative samples, n represents the number of classes, and m*AP* is the mean precision across all detected categories.

## 4. Results and Analysis

### 4.1. The Impact of C2f-GB Module Quantity and Position on Model Performance

In this study, this paper integrated the newly proposed C2f-GB module into the neck network to enhance feature extraction while significantly reducing computational complexity. Given that the YOLOv10 network contains several C2f and C2fCIB modules, this paper conducted a series of experiments to systematically analyze how different quantities and positions of C2f-GB modules affect model performance.

Table 3 shows the impact of varying C2f-GB module counts on model performance. It is evident that as the number of C2f-GB modules decreases, model complexity decreases, resulting in insufficient learning from the training data, which negatively impacts accuracy. However, when all C2fCIB modules in the neck network were replaced with C2f-GB modules, model complexity increased, leading to an improvement in accuracy. As a result, we chose to replace all C2fCIB modules in the neck network with C2f-GB modules.

Table 4 describes the effect of integrating C2f-GB modules at different positions within the network. Group 1 only replaces the first three C2f modules in the backbone network. In Group 2, the last two C2f modules and the final C2fCIB module in the backbone network are replaced. Group 3 includes the replacement of the third C2f module, the last C2fCIB module in the backbone network, and the first C2fCIB module in the neck network. Group 4 replaces the last C2fCIB module in the backbone network and the first two C2fCIB modules in the neck network. Finally, Group 5 represents the model in this experiment, where only the three C2fCIB modules in the neck network are replaced. Experimental results show that when the C2f modules in the backbone network are replaced, the model’s precision and recall are lower. Group 3 has slightly higher precision than Group 5, but other metrics in Group 5 are much better than in Group 3. Overall, integrating C2f-GB in the neck network results in the best model performance.

### 4.2. The Impact of Different Attention Mechanisms on Model Performance

**Figure 11:**
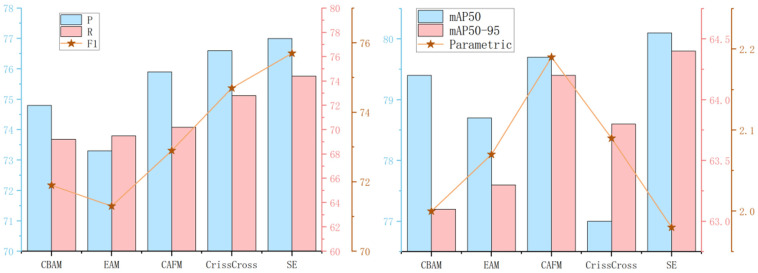
Impact of different attention mechanisms on model performance.

To validate the effectiveness of the proposed channel attention mechanism, this paper integrated it with CBAM, EMA, CAFM, and CrissCross Attention at the same positions in the neck network. The resulting Precision (*P*), Recall (*R*), *F*1 score, parameter count, and m*AP* curves are shown in Figure 11. The results indicate that the model’s performance is optimal when using the SE channel attention mechanism.

Table 5 presents the detailed performance metrics observed after incorporating various attention mechanisms into the model. Notably, SE achieved an impressive mAP50 of 80.1% and a remarkable parameter count of 1.98M, outperforming all other evaluated attention mechanisms. This confirms that the proposed channel attention mechanism can dynamically adjust the attention of each channel, improving the efficiency of information utilization in complex backgrounds.

### 4.3. Ablation Experiment Performance Comparison

To validate the effectiveness of the proposed improvements, this paper conducted ablation experiments on the YOLOv10 model. The results are shown in Table 6. Experiment 1 serves as the baseline network, while Experiments 2–5 each introduce different modifications. As seen in Table 2, removing the largest object detection layer (comparing Experiment 1 with Experiment 2) increased mAP50 from 78.1% to 80.5%, an improvement of 2.4%, and mAP50-95 increased from 63.4% to 64.6%, a 1.2% improvement, while the parameter count decreased by 20.6%.

Subsequently, the introduction of the C2f-GB and BiFPN modules further reduced the model’s weight, although the average precision (mAP) and recall slightly decreased. The addition of the BiFPN module significantly enhanced the model’s ability to extract information, particularly from tomato features, leading to a 1.1% improvement in mAP while reducing the parameter count significantly compared to the baseline.

Finally, after incorporating the SE channel attention mechanism, the modified network demonstrated the ability to intelligently adjust the channel weights, allowing it to focus on key information while filtering out redundant or irrelevant input, thus improving task execution efficiency and accuracy. After this improvement, recall, mAP50, and mAP50-95 increased by 2.1%, 2%, and 1%, respectively, while the parameter count decreased by 26.3%. These results show that the improved network not only enhances precision and recall but also significantly reduces the parameter count.

### 4.4. Comparison of Detection Performance Across Different Models

**Figure 12:**
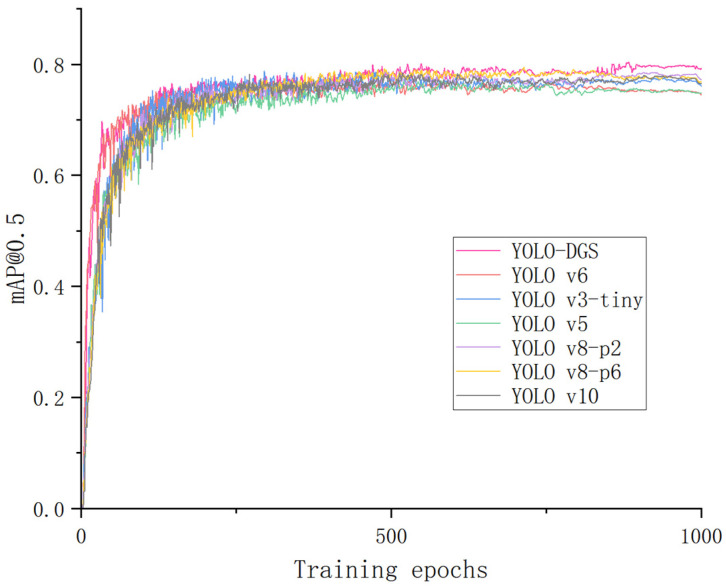
Comparison of average accuracy across different models.

To highlight the effectiveness and innovation of this study, this paper compared the improved YOLO-DGS model with several popular lightweight object detection models. The results are shown in Table 7. YOLO-DGS outperformed YOLOv3-tiny, YOLOv5, YOLOv6, YOLOv8-p2, YOLOv8-p6, and YOLOv10 in recall, with improvements of 3.8%, 3.6%, 1.1%, 4.5%, 2.7%, and 2.1%, respectively. YOLO-DGS also achieved the best mAP50-95 among all comparison models. The average precision comparison between different lightweight models and the improved YOLO-DGS is shown in Figure 12. Furthermore, YOLO-DGS reduced the parameter count by 84.0%, 20.8%, 53.2%, 32.4%, 58.5%, and 26.9%, while the inference speed increased to 416.7 frames per second. Compared to other popular lightweight models, the YOLO-DGS model is not only more compact but also achieves higher accuracy and faster inference speed.

**Figure 13:**
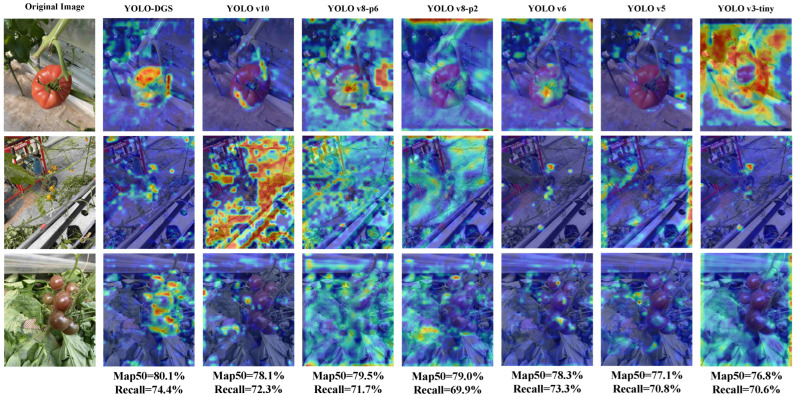
Visualization of model’s focus on key areas of tomato before and after improvement.

This paper used XGrad-CAM [38] for a visual analysis of the model’s performance to evaluate the YOLO-DGS model’s visual focus and attention. The pre- and post-improvement visual attention results for tomato detection are shown in Figure 13. The heatmaps reveal that, for both single-object and multi-object detection tasks, as well as for occluded objects, YOLOv10 shows poor attention to small objects, which reflects the positive effect of removing redundant detection heads. Both YOLOv8 models exhibit attention dispersion and fail to focus on the correct targets. YOLOv6 and YOLOv5 also show insufficient attention to target objects, especially in complex multi-object occlusion scenarios. This highlights the advantage of the dynamic adjustment of channel attention in the improved model, which increases information utilization efficiency. Finally, YOLOv3-tiny suffers from attention dispersion in both single-object and multi-object occlusion cases, and shows a lack of focus on small targets. In contrast, the improved YOLO-DGS model accurately identifies targets in all scenarios, including single-object, small-object, and multi-object occlusion cases. These results demonstrate that YOLO-DGS significantly enhances feature extraction capabilities and is less affected by complex environments.

**Figure 14:**
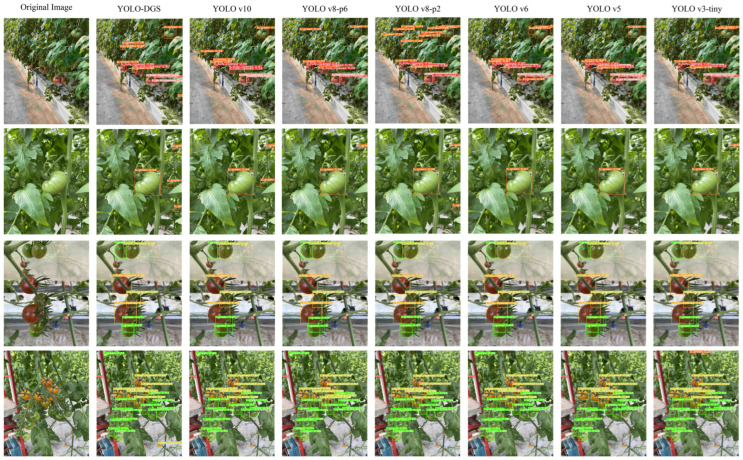
Detection performance of different models.

To further evaluate the model’s performance, this paper conducted a visual comparison of the detection performance of YOLO-DGS and other models under various backgrounds and distances. As shown in Figure 14, YOLOv5, YOLOv6, and YOLOv3-tiny had higher miss detection rates. While YOLOv8 and YOLOv10 models detected most targets, their false detection rates were high, particularly in cluttered backgrounds. For instance, when unripe tomatoes and green leaves appeared in the same frame, YOLOv8-p2 mistakenly identified multiple leaves as unripe tomatoes. At greater detection distances, where the target appeared smaller, only YOLO-DGS successfully detected a distant semi-ripe tomato, while all other models failed. These results indicate that YOLO-DGS, with the proposed improvements, significantly enhances feature extraction capabilities and performs better under varying distances and complex backgrounds, making it more suitable for tomato harvesting in diverse real-world conditions.

## 5. Conclusions

The recognition of tomato maturity is affected by shooting distance, the number of targets, and background interference. Deep learning algorithms, due to their numerous parameters, often suffer from computational complexity and time-consuming processes, which lead to reduced accuracy and efficiency [39,40]. To address the challenges in tomato ripeness recognition, this study proposes an optimized YOLO-DGS algorithm for efficient and accurate real-time tomato detection. The algorithm improves upon YOLO v10 with the following enhancements: (1) redundant object detection layers are removed to enhance the model’s ability to capture the features of specific detection targets and reduce the number of parameters; (2) a novel C2f-GB module is introduced, which uses fewer parameters and computational resources to generate more feature maps, improving the model’s feature extraction capability while reducing both parameter count and computational complexity; (3) a bidirectional feature pyramid network (BiFPN) is incorporated to more effectively capture feature representations at different scales, thereby improving the model’s ability to handle objects of varying sizes and complexities; (4) a channel attention mechanism (SE) is utilized to dynamically adjust channel attention, enhancing information utilization efficiency. To validate the effectiveness of the proposed algorithm, a series of fusion experiments were conducted, comparing the optimized YOLO-DGS model with different numbers of detection heads and various lightweight networks. The experimental results demonstrate that the YOLO-DGS model outperforms others in terms of mean accuracy, parameter count, and inference speed, achieving a better balance between efficiency and precision. This improvement not only enhances the accuracy and efficiency of tomato detection but also provides a lightweight design, making the algorithm more suitable for deployment on embedded devices, ultimately boosting the efficiency of automated tomato harvesting.

Despite certain progress achieved in the method for identifying tomato maturity, this approach still harbors numerous limitations. First and foremost, there is a limitation in terms of variety. Currently, this experiment has only been conducted on common tomatoes and cherry tomatoes. Tomatoes of different varieties exhibit disparities in color, shape, and maturity characteristics. This may impede the applicability of our method to other varieties. For instance, some special tomato varieties do not assume the typical red color when ripe. Take the “Emerald” tomato as an example; it remains green even when fully mature. With the existing color-based recognition method, it is extremely challenging to accurately determine its maturity level. Weather conditions also pose an objective limiting factor. Under overcast days or in insufficient lighting conditions, the color of the captured images will deviate, affecting the judgment of tomato maturity. On the other hand, under direct intense sunlight, the tomato surface will reflect light, causing distortion of color and texture information in the images, thereby reducing the accuracy of recognition.

In summary, although this study has addressed the issue of maturity identification for common tomatoes and cherry tomatoes under general circumstances, in the face of the above-mentioned special cases and variety differences, there is still significant room for improvement. In the future, we will further our research, delving deep into the maturity characteristics of different tomato varieties and optimizing the recognition algorithm to accommodate a wider range of varieties and complex environments. We will also conduct in-depth investigations into these special cases to continuously refine the method for identifying tomato maturity.

## Figures and Tables

**Table 1:** Comparison of detection performance with varying number of detection heads.

Module	P/%	R/%	F1/%	mAP50/%	Parametric/M	FPS/(f/s)	GFLOPs
YOLO v10-1Detect-C2fGB-BiFPN-SE	75.4	70.9	73.0	77.0	1.47	357.1	4.8
YOLO-DGS (2 Detect)	77.0	74.4	75.7	80.1	1.98	416.7	4.9
YOLO v10-C2fGB-BiFPN-SE	76.1	74.1	75.0	80.7	2.70 (2.708201M)	344.8	6.7
YOLO v10-4Detect-C2fGB-BiFPN-SE	76.4	71.3	73.7	78.7	2.92	263.2	12.1

**Table 2:** Configuration settings for the experimental environment.

Type	Configuration
CPU	15 vCPU Intel(R) Xeon(R) Platinum 8474C
GPU	RTX 4090D
Operating system	Windows 11
CUDA	11.1
Programming language	Python 3.8
Deep learning architecture	Pytorch 1.13.1

**Table 3:** Impact of varying number of C2f-GB modules on model performance.

Quantity	P/%	R/%	F1/%	mAP50/%
3	77.0	74.4	75.7	80.1
2	71.9	69.2	70.5	78.2
1	68.3	63.9	66.0	74.3
0	75.2	73.1	74.1	80.5

**Table 4:** Impact of C2f-GB module placement on model performance.

Group	P/%	R/%	F1/%	mAP50/%
1	73.6	64.5	68.9	70.3
2	76.9	63.7	69.7	71.3
3	77.7	69.2	73.2	76.8
4	76.4	69.3	72.7	77.9
5	77.0	74.4	75.7	80.1

**Table 5:** Impact of various attention mechanisms on model performance.

Module	P/%	R/%	F1/%	mAP50/%	mAP50-95/%	Parametric/M
CBAM	74.8	69.2	71.9	79.4	63.1	2.00
EMA	73.3	69.5	71.3	78.7	63.3	2.07
CAFM	75.9	70.2	72.9	79.7	64.2	2.19
CrissCross	76.6	72.8	74.7	77.0	63.8	2.09
SE	77.0	74.4	75.7	80.1	64.4	1.98

**Table 6:** Comparative analysis of the effects of different modules.

Baseline	Order	2Detect	C2f-GB	BiFPN	SE	R/%	mAP50/%	mAP50-95/%	Parametric/M
YOLO v10	1	-	-	-	-	72.3	78.1	63.4	2.70 (2.709380M)
	2	√	-	-	-	73.0	80.5	64.6	2.14
	3	√	√	-	-	72.6	78.7	63.3	2.07
	4	√	√	√	-	70.9	79.2	63.5	1.99
	5	√	√	√	√	74.4	80.1	64.4	1.98

**Table 7:** Comparative performance evaluation of different lightweight detection models.

Model	R/%	mAP50/%	mAP50-95/%	Parametric/M	FPS/(f/s)
YOLO v3-tiny	70.6	76.8	62.0	12.4	76.3
YOLO v5	70.8	77.1	61.2	2.50	60.9
YOLO v6	73.3	78.3	64.1	4.23	78.7
YOLO v8-p2	69.9	79.0	64.0	2.93	73.5
YOLO v8-p6	71.7	79.5	64.3	4.78	76.9
YOLO v10	72.3	78.1	63.4	2.71	370.4
YOLO-DGS	74.4	80.1	64.4	1.98	416.7

## Data Availability

The data presented in this study are available on request from the corresponding author.
